# Infant Botulism: In Search of *Clostridium botulinum* Spores

**DOI:** 10.1007/s00284-024-03828-0

**Published:** 2024-08-13

**Authors:** Richard A. Harris, Haydee A. Dabritz

**Affiliations:** 1 Botulism Reference Service for Canada, Health Canada, Ottawa, ON Canada; 2 Infant Botulism Treatment and Prevention Program, California Department of Public Health, Richmond, CA USA

## Abstract

Infant botulism is now the most common form of human botulism in Canada and the United States. Infant botulism is a severe neuroparalytic disease caused by ingestion of the spore-forming neurotoxic clostridia, including *Clostridium botulinum* that colonize the large intestine and subsequently produce botulinum neurotoxin in situ. It has been over a century since the first surveys documenting the ubiquitous prevalence of *C. botulinum* in soils around the world. Since then, honey has been identified as the only well-known risk factor for infant botulism despite a multitude of international environmental surveys isolating *C. botulinum* spores from ground soil, aquatic sediments, and commonly available infant foods. Associations of infant botulism cases with confirmed sources of *C. botulinum* exposure have primarily implicated outdoor soil and indoor dust, as well as commonly ingested foods including honey, dry cereals, and even powdered infant formula. Yet the origin of infection remains unknown for most infant botulism cases. This review summarizes the various surveys from around the world for *C. botulinum* in environmental soils and sediments, honey, and other infant foods, as well as laboratory-confirmed associations with documented infant botulism cases. Additional factors are also discussed, including the composition of infant gut microbiota and the practice of breastfeeding. We make several recommendations to better identify sources of exposure to *C. botulinum* spores that could lead to effective preventive measures and help reduce the incidence of this rare but life-threatening disease.

**Supplementary Information:**

The online version contains supplementary material available at 10.1007/s00284-024-03828-0.

## Introduction

*Clostridium botulinum* is a Gram-positive, spore-forming, obligate anaerobic bacterium that causes the neuroparalytic disease of botulism by producing botulinum neurotoxin (BoNT). BoNTs cause muscle paralysis by cleaving pre-synaptic vesicle proteins responsible for releasing acetylcholine from cholinergic nerve terminals at neuromuscular junctions. Some rare pathogenic strains of closely related species including *C. baratii*, *C. butyricum*, *C. argentinense,* and *C. sporogenes*, can also produce BoNTs [1]. *C. botulinum* is categorized into four different physiologically and genetically distinct groups (I-IV) that express at least eight different serotypes of BoNT (A-H), including the putative novel toxin BoNT type X. Groups I and II *C. botulinum* are most associated with human botulism and express BoNT types A, B, F, and B, E, F, respectively [2]. The proteolytic group I *C. botulinum* can form spores that are highly heat resistant and have a higher optimal growth temperature (35 °C) than the non-proteolytic group II *C. botulinum*, which form heat-sensitive spores and can grow at a lower minimum temperature (4 °C). Group III *C. botulinum* expresses BoNT types C and D that are associated with animal botulism [3], while group IV *C. botulinum* expresses BoNT type G that has been implicated in human disease but is exceptionally rare [4].

Botulism is categorized by the different routes of exposure to BoNTs. Foodborne botulism is caused by ingestion of foods contaminated with *C. botulinum* that have been permitted to grow and produce BoNT in the food. Thus, foodborne botulism is an intoxication caused by ingestion of preformed BoNT. Adult colonization botulism and wound botulism are caused by *C. botulinum* colonization of the gastrointestinal tract and infected wounds, respectively, with production of BoNTs in situ. Adults with healthy gastrointestinal tracts are resistant to *C. botulinum* colonization and may unknowingly ingest spores on a regular basis. Infant botulism is now the most common form of botulism in Canada and the United States [5, 6], and is caused by colonization of the gastrointestinal tract in infants predominantly less than one year of age. Infants are particularly susceptible to colonization by *C. botulinum* at an age between three to five months old [7] that coincides with perturbations in gut microbiota after weaning from breast milk to solid foods [8], although cases have been described at younger than one week old [9, 10]. Infant botulism is most associated with group I *C. botulinum* types A and B, yet cases have also been caused by group II *C. botulinum* type E [11], as well as *C. butyricum* type E [12–16], and *C. baratii* type F [10, 17–19]. In a single instance, group III *C. botulinum* type C causing infant botulism has been described [20].

In contrast to a foodborne botulism outbreak, in which multiple patients can be traced to a single-shared food source, infant botulism is caused by the ingestion of *C. botulinum* spores commonly found in the environment and occurs sporadically as individual cases. Over a century has passed since the pioneering surveys by Meyer and colleagues in 1922 that found viable *C. botulinum* spores in the ground soils, aquatic sediments, and food products in the United States, Canada, Europe, and China [21–25]. Since then, environmental surveys from around the world have isolated *C. botulinum* spores from soils and sediments, as well as foods commonly fed to infants such as honey, dry cereals, fruits, and vegetables, and even powdered infant formula [26, 27]. Honey is the most well-known risk factor for infant botulism [28]. Household dust and outdoor soils that were phylogenetically identical to patient isolates have also been confirmed as sources of infection [29]. The largest case–control study of infant botulism in the United States to date, conducted from 1976 to 1983, identified the following risk factors for infant botulism associated with illness hospitalization: breastfeeding, the occurrence of less than one bowel movement per day, ingestion of honey or corn syrup, dust exposure, and residence in a windy area [30]. Yet source attribution for infant botulism remains a significant challenge. In Canada, from 1979 to 2019, only 6% of cases were attributed to a laboratory-confirmed source, all of which were from honey [5].

This article summarizes the findings of published *C. botulinum* surveys including infant foods and environmental sources from around the world. The eligibility criteria included primary research articles from all peer-reviewed journals from all countries with no limit to the year of publication. Information sources included PubMed® (National Institutes of Health, USA) and Google Scholar. By creating a complete and unified summary of available information, we identify several gaps in knowledge where future investigations could reveal common food or environmental reservoirs where infant exposure to *C. botulinum* is most likely to occur, thereby leading to simple and effective preventive measures to reduce the incidence of this life-threatening childhood disease.

## Discussion

### Honey Surveys

Honey was recognized as a potential source of *C. botulinum* spores in 1976 when infant botulism was first described by Midura and Arnon [31]. By 1979, several case associations had been made linking *C. botulinum* isolates from patients’ clinical specimens to honey isolates, and several different surveys had also recovered *C. botulinum* from retail honey samples [32–36]. In 1981, the American Academy of Pediatrics issued a policy statement advising against feeding honey to infants [37], which has since resulted in a significant decline in infant botulism cases associated with honey ingestion [38]. In total, at least 70 published surveys, independent of associations with infant botulism cases, have been conducted globally from 1978 to 2023 on honey sampled directly from apiaries or retail sources and are listed in Supplementary Table 1. Overall, *C. botulinum* was found in 173/4,310 (4%) of honey samples from around the world. Most of the isolates recovered were type A (67/173, 39%) and type B (63/173, 36%). This agrees with the observation that *C. botulinum* type A and B spores are most associated with mainland soils and are accumulated by bees during nectar collection [39]. In addition, BoNT type was unknown in 33/173 (19%) specimens. Type E and F were recovered in 5/173 (3%) specimens each, and a type AB (dual-toxin-producing strain) was recovered in 1/173 (< 1%) specimen. Honey poses no risk for foodborne botulism due to its low water activity (0.5–0.65) that inhibits the growth of vegetative *C. botulinum* cells (> 0.94 required) [40], and as such no instance of preformed BoNT has ever been reported in honey.

Honey produced from different countries had widely differing prevalences of *C. botulinum* contamination. Denmark was found to produce honey at the highest prevalence with 29/112 (26%) of samples testing positive from a single survey [41]. In the United States, *C. botulinum* was found in 42/587 (7%) of honey samples from nine different surveys. In Germany, only 1/354 (< 1%) of honey samples tested positive from three different surveys and honey from the UK and Canada contained 0/122 and 0/112 positive samples, respectively. In 1998, a case of infant botulism from Norway was linked to honey containing *C. botulinum* type A from Argentina [42]. Honey produced in Argentina has a *C. botulinum* prevalence of 12/318 (4%) and high proportion of type A (9/12, 75%) when six independent surveys are combined. Several studies tested small batches of retail honey produced from around the world. For example, in 2002 a survey tested retail honey sold in Finland that was produced in 16 different countries spanning three continents [43]. This investigation found that honey tested in small sample sizes from some countries had a high prevalence of contamination, such as Argentina (3/4, 75%), Australia (2/7, 29%), Spain (2/8, 25%), Italy (1/2, 50%), Cuba (1/5, 20%), and France (1/5, 20%). However, a much lower prevalence was found after combining multiple surveys, or incorporating surveys performed at higher scales. For example, when combining all three surveys on honey produced in Italy, the incidence was 3/139 (2%) overall, and combining the two surveys on honey produced in France the incidence was 1/95 (1%) overall. This demonstrates the importance of incorporating a higher sample size for individual surveys or combining multiple surveys together when inferring the prevalence of *C. botulinum* spores in a given food or environmental sample.

### Other Infant Food Surveys

In addition to honey as a source of spores, other infant foods such as corn syrup, dry cereals, and powdered infant formulas, were surveyed for the presence of *C. botulinum*. In total, at least 18 different surveys from 1922 to 2020 were conducted on retail infant food items and are summarized in Supplementary Table 2. In 1982, a survey of retail corn syrups sold in Washington, DC, isolated *C. botulinum* from 8/40 (20%) of samples tested, and a follow-up survey found 5/961 (< 1%) of samples tested positive [44]. Corn syrup manufacturers revised their production methods in the 1980s and a subsequent large-scale survey of retail corn syrups in 1991 failed to isolate *C. botulinum* from 738 samples tested, which led the authors to conclude that corn syrup is unlikely to be a source of infection for infant botulism [45, 46]. In 1992, a survey in Japan recovered *C. botulinum* from raw, but not refined, sources of sugar sampled directly from producers, including sugar for apiculture (2/56, 4%), raw sugar (2/22, 9%), molasses (2/5, 40%), brown sugar (2/41, 5%), and corn syrup (1/16, 6%) [47]. A series of interesting surveys in 2008 and 2009 from Argentina found *C. botulinum* in chamomile (15/200, 8%) and linden flower (3/100, 3%) tea leaves when sampled directly from producers, yet all 100 samples of linden flower tested from retail sources were negative [48, 49]. Retail samples of dry cereals were found to be negative when tested in surveys from 1979 (0/30), from 1982 (0/90), and also from 1983 (0/12). However, in 1988, a survey from Canada isolated *C. botulinum* in one sample of retail dry rice cereal (1/40, 3%) [50]. The contamination was only found in 1/3 replicates and a follow-up investigation of three cereals of the same lot was all negative.

In 2005, a case of infant botulism in the UK was linked to an opened can of powdered infant formula [26]. An opened container of dried rice pudding also contained *C. botulinum*, but of a different BoNT type to the patient. A follow-up survey of retail powdered infant formulas isolated *C. botulinum* from 1/15 (7%) of samples from the same batch, but the isolate was of a different genetic profile [27]. In response to this incident, an ad hoc risk assessment was conducted in 2006 by an advisory committee on behalf of the UK Food Standards Agency, which concluded that the risk of infant botulism from powdered infant formulas was low due to the stringent hygiene practices employed during production and a low calculated prevalence of spores [51]. In 2008, the Codex Alimentarius Commission deemed that *C. botulinum* is not considered a hazard in powdered infant formula [52], and in 2014, the International Commission on Microbiological Specifications for Foods recommended that powdered infant formulas and dairy-based ingredients for infant formulas not be tested for *C. botulinum*, but that routine testing for sulfate-reducing clostridia be used to determine adherence to Good Hygiene and Manufacturing Processes [53]. There are currently no clostridial standards for powdered infant formula in the USA or Canada. Retail powdered infant formulas from the United States were previously tested by surveys in 1979 and 1982, which failed to recover *C. botulinum* in 50 and 100 samples tested, respectively [33, 44]. More recently, a 2006–2007 survey from California did not recover *C. botulinum* from nine retail powdered infant formula samples and 30 opened cans from patients’ households, although non-pathogenic clostridia were recovered from both retail and home samples [54]. Finally, in 2017, a *C. botulinum* isolate from an opened powdered infant formula was genetically linked to an isolate from the enema fluid of an infant botulism patient from China [55].

Fresh fruits and vegetables are a well-known source of *C. botulinum* spores due to their growth near soil and associations with foodborne botulism outbreaks. Yet, this category of foods can be prepared for infants without cooking. Even the process of boiling vegetables to prepare baby purees may not be sufficient to destroy all heat-resistant group I *C. botulinum* spores that are most often associated with cases of infant botulism. The first two surveys of fruits and vegetables were conducted in 1922 in the United States, which isolated *C. botulinum* from 54/431 (13%) to 33/122 (27%) of samples tested, including carrots (2/18, 11%), string beans (14/44, 32%), peas (3/51, 5%), and tomatoes (2/24, 8%) [21, 22]. A survey in 1975 from Canada isolated *C. botulinum* from all 12/12 (100%) of fresh commercial button mushrooms (*Agaricus campestris*) [56]. Since then, the prevalence of *C. botulinum* in this category of foods has been poorly investigated and limited to the context of foodborne botulism. In 1995, a survey from the United States found that 4/1,118 (< 1%) of pre-packaged vegetables tested positive for *C. botulinum*, including one isolate each for shredded cabbage, chopped green pepper, Italian salad mix, and escarole salad mix [57]. A PCR-based screening of raw vegetables used for canned purees in 2001 from France found 0/37 testing positive for *C. botulinum* [58]. A study in 2012 from France recovered *C. botulinum* type B from 1/128 (0.8%) of raw carrots and 1/188 (0.5%) of raw green beans used for canning [59]. More recently, a high prevalence (24/74, 32%) of *C. botulinum* from both groups I and II and types A, B, E, and F was detected in retail vegetarian sausages by a survey in 2020 from Finland [60]. In the context of food manufacturing, it is simply assumed that viable *C. botulinum* spores may be present in raw fruits and vegetables, and efforts are taken to destroy them or prevent their growth. For infant botulism, this category of foods represents a significant risk that has yet to be fully investigated in recent years.

### Environmental Surveys

The first person to suggest that *C. botulinum* is widely distributed in nature was G.S. Burke in 1919, who recovered isolates from various fruits, vegetables, insects, and other environmental samples in five different locations across California [61]. In 1922, four different landmark surveys were conducted by Meyer and colleagues investigating the prevalence of *C. botulinum* spores in the mainland soils and aquatic sediments of the United States, Canada, Europe, and China [21–25]. At least 73 different environmental surveys from 37 countries have been conducted from 1919 to 2023 and are summarized in Supplementary Table 3. In total, viable *C. botulinum* spores were recovered from 4,655/36,793 (13%) of environmental samples. Mainland soils were extensively tested due to concerns of foodborne botulism from fruits and vegetables grown on cultivated farmlands. Of the 51 surveys conducted on mainland soils worldwide, *C. botulinum* was recovered from 2,498/19,289 (13%) of samples tested. *C. botulinum* was also recovered from 1,314/11,041 (12%) of coastal sediments across 32 surveys and 761/5,151 (15%) of inland sediments across 21 surveys. One study in 1998 from Finland reported a high prevalence of *C. botulinum* type E in sea water (37/42, 88%) and fresh water (34/56, 61%) sampled from the Baltic Sea and Finnish mainland, respectively [62]. Interestingly, the prevalence of *C. botulinum* spores is lower when sampled from indoor household dust. Three small-scale studies in 1979 from California recovered *C. botulinum* from indoor dust in 1/19 (10%), 2/31 (7%), and 1/46 (2%) of samples tested [33, 34, 36]. More recently, a 2023 study from Canada did not detect *C. botulinum* in 963 samples of household vacuum cleaner dust across 13 major Canadian cities [63]. These limited surveys suggest that exposure to *C. botulinum* is less likely indoors unless spores are carried in from an outside source. A survey in 1985 isolated *C. botulinum* from 3/4 (75%) of the outdoor soil samples from the father’s worksite and all four samples of dirt from the father’s shoes, indicating a likely vector of transfer from outdoors to indoors [64]. Indeed, several cases of infant botulism have been matched by BoNT type to isolates of both indoor dust and outdoor soil from the surrounding environment [34].

The factors affecting the geographic distribution of *C. botulinum* spores in different environmental niches are poorly investigated, yet some consistent trends have emerged. Overall, mainland soils were most associated with types A (1,062/2,498, 43%) and B (809/2,498, 32%), while aquatic sediments, sea water, and fresh water were most associated with type E (1,851/2,152, 86%). In some instances, types A and B were recovered from aquatic sediments (211/2,075, 10%) and type E was isolated from mainland soils (284/2,498, 11%). *C. botulinum* type F was isolated from both mainland soils (61/2,498, 2%) and aquatic sediments (13/1,314, 1%). Dual-toxin-producing clostridia were only obtained from mainland soil samples, including type Ab (21/2,498, < 1%), type Af (26/2,498, 1%), and on one occasion type Bf (1/2,498, < 1%). Surveys in 1922 and 1978 from the United States found a clear disparity in the geographic distribution of type A and B spores [21, 65]. Type A spores were predominantly isolated west of the Mississippi river in neutral or alkaline soils with lower organic content. Type B spores were more commonly found in the eastern states in more acidic soils with higher levels of organic content. This distribution is reflected in the BoNT type of *C. botulinum* associated with infant botulism patients in the USA [6]. Worldwide, type A and B spores were more commonly found in the mainland soils of temperate countries, including Argentina (564/2,732, 21%), Brazil (67/314, 21%), China (577/7,378, 8%), Republic of Georgia (40/258, 16%), Taiwan (75/134, 56%), Italy (7/520, 1%), the UK (48/711, 7%), Hawaii, USA (7/19, 37%), and the continental USA (398/2,788, 14%). The psychrotrophic group II *C. botulinum* type E is known as a water-dwelling organism that is often linked to foodborne botulism outbreaks in marine mammals and fish [66]. Overall, *C. botulinum* type E was isolated at a high prevalence from the aquatic sediments of coastlines in northern countries, including Alaska, USA (92/292, 32%), Canada (214/999, 21%), Greenland (18/21, 86%), Denmark (337/629, 54%), Norway (8/12, 67%), Sweden, (236/312, 76%), and Finland (92/137, 67%). Type E spores were also found on the Pacific coastline (224/662, 34%), Atlantic coastline (11/341, 3%), and Gulf coastline (5/717, < 1%) of the continental United States.

The original 1922 surveys from the United States found a higher prevalence of *C. botulinum* spores in undisturbed soils that were still in their natural state (150/413, 36%) than in soils that had been disturbed by people through cultivation or urbanization (215/904, 24%) [21, 22]. This was also observed in Canada with undisturbed soils showing a greater prevalence (23/61, 38%) than disturbed soils (4/30, 13%) [25]. However, this finding has not been consistent internationally or regionally within the United States. In 1922, the same authors failed to recover *C. botulinum* from seven undisturbed soils from the UK, yet were successful for 9/57 (16%) of disturbed soils [23]. A 1946 survey from New York State found that undisturbed soils had a lower prevalence (3/60, 5%) of *C. botulinum* than disturbed soils (26/178, 15%) [67]. In 1980, a survey from Denmark also found a lower prevalence in undisturbed soils (5/38, 13%) than in disturbed soils (13/37, 35%) [68]. Finally, in 2005 a large-scale survey from Argentina found a lower prevalence of spores in undisturbed soils (108/661, 16%) than disturbed soils (256/722, 36%), although this trend was reversed in the central region of Argentina that includes the dense urban city of Buenos Aires, where *C. botulinum* was isolated from 89/397 (22%) of undisturbed soils and 31/88 (35%) of disturbed soils [69]. In total worldwide, 27 studies have isolated *C. botulinum* spores from 484/2,211 (22%) of undisturbed soils and 32 studies have isolated spores from 967/5,140 (19%) of disturbed soils, suggesting that this is not a geographically persistent trend. It is most likely that spores of *C. botulinum* are simply ubiquitous in nature worldwide and may be incorporated into cultivated farmland or urban areas through inadvertent human development.

### Case-Confirmed Associations

Case study investigations, in which isolates of *C. botulinum* from the intestinal contents of patients with infant botulism matched the BoNT type of isolates recovered from ingested food or the local environment, are listed in Supplementary Table 4. Overall, outdoor soil samples were found to contain *C. botulinum* in 39/44 (89%) of associated cases, while indoor dust was positive in 26/156 (17%) of associated cases, and honey was positive in 52/77 (68%) of associated cases. Powered infant formula was associated with two cases, one from the UK in 2005 and one from China in 2017 [26, 55]. A dry rice cereal was associated with one case from China in 2015 [70]. Chamomile herbs were associated with a single case from Portugal in 2012, although ingested honey was also found to contain *C. botulinum* with the same BoNT type [71]. Finally, contaminated well water was associated with one case from Japan in 2014 [72], and water from a pet turtle aquarium was found to contain *C. butyricum* type E associated with two closely related cases from Ireland in 2015 [73]. In some instances, such as a survey conducted in 1979 from the United States, only a small portion of sampled honey (6/17, 35%) or dust (4/85, 5%) recovered *C. botulinum* that matched clinical isolates from the patients residing in associated households [34]. In other studies, such as a survey in 1986 from the United States, all 11 (100%) outdoor soils and 2/3 (67%) of indoor dust samples contained *C. botulinum* that matched the BoNT type of three geographically related infant botulism cases from a small town in Colorado [64].

These targeted surveys and case study investigations demonstrate that, in most instances, the laboratory-confirmed sources of *C. botulinum* exposure are from expected sources. Honey, outdoor soils, and indoor dust accounted for 114/121 (94%) of all verified sources of spores. Of the case studies investigating contaminated honey, 43/49 (88%) occurred before the year 2000, which indicates a collective recognition of honey as a risk factor for infant botulism. The most recent case associated with honey occurred in Israel in 2019 involving a contaminated honey-cake [74]. Outdoor soils were more often found to be a source of exposure (39/120, 33%) than indoor dust (26/120, 22%), which is expected considering that *C. botulinum* is commonly known as a soil-dwelling organism ubiquitous in nature that would need to enter the household via heating/cooling vents or be tracked inside on shoes, clothing, or by pets. One important note is that most of these case associations were established at the level of BoNT type. Comparative genotyping is only rarely performed to confirm these isolates as the definitive source of infection. A study published in 2014 from California performed genetic epidemiological testing at a large scale and found that *C. botulinum* isolates from patients’ clinical samples matched to the same genetic clade as 11/14 (79%) of environmental isolates and all 10 (100%) of honey isolates, strongly suggesting that these isolates were the sources of infection [29].

### Additional Factors

A well-known risk factor for infant botulism is the age between three and five months old [7] that coincides with substantial changes to intestinal bacterial taxa after weaning off breast milk onto solid foods [8]. The same shift in microbial ecology is not as substantial in formula-fed infants [75]. Initially the infant gut microbiome has low species diversity and high variability among individuals [76, 77], yet progresses within the first few years of life toward a stable adult profile by a variety of factors, including lifestyle and diet [78]. The first indications that the gut microbiome protects against *C. botulinum* colonization were from studies in mice in 1979 and 1982 demonstrating a susceptibility to infection in germfree mice and mice treated with antibiotics compared to normal mice [79–81]. Interestingly, a study from Utah published in 1980 of 12 infant botulism patients and 87 controls discovered that 20/87 (23%) of control infants were “asymptomatic carriers” of *C. botulinum* in stool samples tested, of which two also contained BoNT [82]. This finding has yet to be replicated. In 1985, a predominance of Enterobacteriaceae was observed in the stools of all seven infant botulism patients investigated [64]. In 2015, a case–control study found that infants with laboratory-confirmed botulism displayed a significantly higher abundance of Proteobacteria, including Enterobacteriaceae, and a reduction in Lactobacilli and Firmicutes (the phylum that includes clostridia), as compared to infant patients without laboratory confirmation of botulism [83]. Enrichments in Enterobacteriaceae can be triggered by host-mediated inflammatory responses to infection, chemical agents, or genetics, and significantly reduce the abundance of healthy resident bacteria [84]. Recently, the gut microbiota of an infant botulism case with particularly long-lasting fecal excretion of *C. botulinum* and BoNT was characterized, and revealed a persistently high abundance of Bifidobacteria that usually decreases by one year of age following the introduction of solid foods [85]. Interestingly, two bifidobacterial species *B. breve* and *B. bifidum* colonized the gut at later stages of the disease correlating with the clearance of *C. botulinum*. Bifidobacteria are prominent in the neonatal infant gut and have been shown to inhibit colonization of a broad range of enteropathogens [86], as well as prevent BoNT absorption and *C. botulinum* growth in vitro [87, 88]. *Lactobacillus paracasei* subsp. *paracasei* was also shown to inhibit *C. botulinum* growth and toxin production in vitro and its administration prior to infection with *C. botulinum* enhanced survival time in mice [89]. While these preliminary studies suggest a role for probiotics in preventing infant botulism, undertaking a prospective study to evaluate the effect of probiotic supplementation would be impractical due to the rarity of disease. Administration of probiotics after disease onset is unlikely to alter the clinical course or length of hospitalization since the negative effects of toxin on the nervous system have already occurred by the time of diagnosis. Probiotics during recovery could help establish healthy microbiota that may protect against other intestinal pathogens, strengthen the intestinal mucosal barrier, reduce inflammation, and modulate the immune response in a positive manner [90]. Further characterization of the infant gut microbiome could help predict the risk of colonization at a distinct age of susceptibility. It is important to note that, unless another infectious organism is detected, antibiotic use for infant botulism patients, especially aminoglycosides, is not recommended due to lysing of vegetative *C. botulinum* cells that could cause a release of additional BoNT and worsen the patient’s paralysis [91].

Breastfeeding was first identified as a potential resilience factor in 1980 when it was found that exclusively breastfed infants developed symptoms at an older age (typically six months or older), while formula-fed infants were hospitalized at younger ages and developed more severe and fulminant illness [92]. This finding was recently confirmed by analysis of data from California spanning 40 years: breastfed patients were more than twice as old at symptom onset as formula-fed patients [38]. The authors suggested that breast milk may influence the composition of the infant gut microbiome to delay *C. botulinum* colonization or prevent the absorption of BoNT through the intestinal epithelium. This effect could be mediated by the predominance of gut bacteria highly adapted to process milk oligosaccharides, such as Bifidobacteria [7], while formula-fed infants are already exposed to more diverse nutrients and develop a microbial profile more closely resembling the adult microbiome earlier [75]. However, the role of breast milk in infant botulism is controversial. A seven-year case–control study conducted from 1976 to 1983 in California found a greater percentage of infant botulism cases were still being breastfed at symptom onset than age-matched healthy controls [30]. A bivariate analysis identified breastfeeding at birth, breastfeeding at onset of symptoms, and number of breastfeeds per day as risk factors for illness hospitalization compared to healthy neighborhood or county controls. One possible explanation for this unexpected finding is that breastfed infants can survive colonization by *C. botulinum* long enough to be diagnosed in a hospital, while non-breastfed infants succumb to fulminant disease, remain as unrecognized cases, and may be classified as cases of Sudden Infant Death Syndrome [36, 93]. A subsequent multivariate analysis revealed that breastfeeding was only a predisposing factor for infants older than two months of age. This agrees with previous epidemiological studies in 1985 and 2014 that identified breastfeeding as being associated with infant botulism cases [64], and as being a significant risk factor for infant botulism at greater than two months of age [29]. There is no evidence that *C. botulinum* or BoNTs are passed through breast milk to the infant. Therefore, it is generally thought that breastfeeding slows down colonization of the infant microbiome with *C. botulinum,* delays the timing of illness onset, and prevents the progression of life-threatening illness when an infant is under constant exposure to *C. botulinum* spores. Thus, paradoxically, breastfeeding has been associated with an increased risk of infant botulism overall [94], and further investigation is warranted to elucidate the mechanisms that mediate these effects.

### Limitations

There are several limitations to these surveys and case associations. First, the isolation of *C. botulinum* is traditionally determined by the presence of the BoNT in liquid cultures and through neutralization assays using monoclonal antibodies in rodents. The original surveys by Meyer and colleagues in 1922 predate the knowledge of types E (1936), F (1960), and G (1970) [40]. Serological-based identification may also fail due to low concentrations of BoNT in the sample, in which neutralizations would be difficult to distinguish from untreated assays [68, 95]. Overall, a significant percentage of honey isolates (33/174, 19%), other food isolates (68/143, 48%), and environmental isolates (340/4,627, 7%) were of unknown BoNT type. Identification by BoNT type without species determination also precludes the possibility that the organisms identified were neurotoxigenic clostridia other than *C. botulinum*, such as *C. baratii* type F or *C. butyricum* type E, which have been linked to previous cases of infant botulism. Furthermore, only rarely were isolates of *C. botulinum* types B or F differentiated as group I or group II by proteolytic digestion of meat pellets in culture media. Traditional intensive heat treatments used for the selection of *C. botulinum* likely destroyed group II spores entirely [96]. Several factors in environmental samples could have also prevented the growth and identification of neurotoxigenic clostridia, including the presence of bacteriophages, bacteriocins, and competing microorganisms that are common in soil or sediments. In addition, the isolation of *C. botulinum* spores from food matrices such as honey and other syrups can be challenging and requires the use of a suitable method to avoid false negatives [39]. Modern molecular methods of detecting *C. botulinum*, including PCR targeting of BoNT genes from established enrichment cultures allow for highly sensitive and specific epidemiological investigations [60]. Finally, due to the predominant association of *C. botulinum* types A, B, E, and F with human illness, sampling instances of types C, D, and G were not reported in this summary. In 1993, Karen Dodds nicely summarized the food and environmental surveys that have isolated *C. botulinum* types C and D from around the world [40].

## Conclusions

Based on the findings in this summary, we can make several recommendations for future investigations to address gaps in knowledge that could aid in discovering environmental sources of *C. botulinum* spores and propose some simple interventions that could help prevent infant botulism infections in the future. First, it is apparent that *C. botulinum* spores are ubiquitous in nature around the world, yet the prevalence in household dust, where exposure to infants is most likely to occur, is inadequately characterized. Several case associations linking *C. botulinum* isolated from the stool of infant botulism patients to isolates from household dust have demonstrated the apparent risk of infection in this setting. It is likely that geographic areas with a high prevalence of *C. botulinum* spores in outdoor soils also correspond to a high prevalence in indoor dust. Therefore, soil surveys to identify highly contaminated areas that also correspond to a high prevalence indoors in residential neighborhoods could be coupled to public awareness for parents with infants in the high-risk age range to recognize symptoms of illness at early stages. Second, fresh fruits and vegetables that are commonly ingested by infants with minimal processing have not been adequately investigated. This potential source of *C. botulinum* spores represents a diverse group of foods that are widely available to infants and have been previously documented to contain *C. botulinum* spores. A large-scale survey could identify fresh fruits and vegetables that are more commonly contaminated with spores. Results from such a survey would help inform parents with infants in the high-risk age range to avoid feeding these foods to infants. This same survey would also help identify foods that are at high risk of causing foodborne outbreaks from improper home canning or home jarring. Instances where neurotoxigenic clostridia are isolated from food or environmental samples should be followed up with characterization by proteolysis from a pure culture (to determine group I vs. group II) or whole-genome sequencing to determine species (*C. botulinum* or otherwise). Third, parents should be aware that when an infant is transitioning from breast milk to formula, or introducing solid foods, a window of vulnerability to infant botulism may exist. At this time in an infant’s life, parents should be aware that several days of constipation followed by weakness and poor feeding are the hallmarks of infant botulism, and they should seek medical advice from a pediatrician. In addition, the excretion of BoNT in the feces of recovering patients with IB may represent a risk to other infants in daycare or other settings because of possible contact with fecally soiled material [97]. Parents, caregivers, and clinicians should follow good hygienic practices that include carefully changing and disposing of fecally soiled diapers, disinfecting the changing surface, and thoroughly washing hands. Finally, enhanced communication with public health officials and hospital staff is essential to coordinate the collection and testing of environmental and food samples in cases of laboratory-confirmed infant botulism. Case-by-case investigations could help identify recurring patterns of infection over the course of a long time period and could justify recommendations to avoid any implicated foods commonly fed to infants. In instances where the source of *C. botulinum* spores is soil or dust near the infant’s residence, exposure may be unavoidable. Clinicians and parents will need to remain vigilant regarding the symptoms of infant botulism, especially in regions where infant botulism is known to occur more frequently. These future endeavors could identify commonly encountered sources of *C. botulinum* spores that may lead to simple but effective preventive measures against this life-threatening childhood disease.

## Supplementary Information

Below is the link to the electronic supplementary material.

Supplementary file1 (XLSX 18 kb)

Supplementary file2 (XLSX 16 kb)

Supplementary file3 (XLSX 27 kb)

Supplementary file4 (XLSX 17 kb)

## Data Availability

Not required for this study.

## Code Availability

Not required for this study.

## References

[1] Smith T, Williamson CHD, Hill K, Sahl J, Keim P (2018) Botulinum neurotoxin-producing bacteria. Isn’t it time that we called a species a species? mBio. 9:1469 10.1128/mBio.01469-18 PMC6156192 30254123

[2] Hauschild A (1989) Clostridium botulinum. Foodborne Bacterial Pathogens. New York, Marcel Dekker Inc, Doyle M, p 111

[3] Meurens F, Carlin F, Federighi M, Filippitzi M, Fournier M, Fravalo P, Ganière J, Grisot L, Guillier L, Hilaire D et al (2022) *Clostridium botulinum* type C, D, C/D, and D/C: an update. Front Microbiol 13:1099184. 10.3389/fmicb.2022.1099184 36687640 10.3389/fmicb.2022.1099184 PMC9849819

[4] Sonnabend O, Sonnabend W, Heinzle R, Sigrist T, Dirnhofer R, Krech U (1981) Isolation of *Clostridium botulinum* type G and identification of type G botulinal toxin in humans: report of five sudden unexpected deaths. J Infect Dis 143:22–27. 10.1093/infdis/143.1.22 7012244 10.1093/infdis/143.1.22

[5] Harris R, Tchao C, Prystajecky N, Cutler J, Austin JW (2021) A summary of surveillance, morbidity and microbiology of laboratory-confirmed cases of infant botulism in Canada, 1979–2019. Can Commun Dis Rep 47:322–328. 10.14745/ccdr.v47i78a05 34421389 10.14745/ccdr.v47i78a05 PMC8340675

[6] National Botulism Surveillance Summary, 2018 | Botulism | CDC. (2022) https://www.cdc.gov/botulism/php/national-botulism-surveillance/2018.html. Accessed Apr 3,2023.

[7] Koepke R, Sobel J, Arnon SS (2008) Global occurrence of infant botulism, 1976–2006. Pediatrics 122:73. 10.1542/peds.2007-1827 10.1542/peds.2007-1827 18595978

[8] Koenig JE, Spor A, Scalfone N, Fricker AD, Stombaugh J, Knight R, Angenent LT, Ley RE (2011) Succession of microbial consortia in the developing infant gut microbiome. Proc Natl Acad Sci U S A 108:4578–4585. 10.1073/pnas.1000081107 20668239 10.1073/pnas.1000081107 PMC3063592

[9] Thilo EH, Townsend SF, Deacon J (1993) Infant botulism at 1 week of age: report of two cases. Pediatrics 92:151–153. 10.1542/peds.92.1.151 8516064

[10] Barash JR, Tang TWH, Arnon SS (2005) First case of infant botulism caused by *Clostridium baratii* type F in California. J Clin Microbiol 43:4280–4282. 10.1128/JCM.43.8.4280-4282.2005 16082001 10.1128/JCM.43.8.4280-4282.2005 PMC1233924

[11] Lúquez C, Dykes JK, Yu PA, Raphael BH, Maslanka SE (2010) First report worldwide of an infant botulism case due to *Clostridium botulinum* type E. J Clin Microbiol 48:326–328. 10.1128/JCM.01420-09 19906896 10.1128/JCM.01420-09 PMC2812300

[12] Abe Y, Negasawa T, Monma C, Oka A (2008) Infantile botulism caused by *Clostridium butyricum* type E toxin. Pediatr Neurol 38:55–57. 10.1016/j.pediatrneurol.2007.08.013 18054696 10.1016/j.pediatrneurol.2007.08.013

[13] Dykes JK, Lúquez C, Raphael BH, McCroskey L, Maslanka SE (2015) Laboratory investigation of the first case of botulism caused by *Clostridium butyricum* type E toxin in the United States. J Clin Microbiol 53:3363–3365. 10.1128/JCM.01351-15 26246485 10.1128/JCM.01351-15 PMC4572528

[14] Fenicia L, Da Dalt L, Anniballi F, Franciosa G, Zanconato S, Aureli P (2002) A case of infant botulism due to neurotoxigenic *Clostridium butyricum* type E associated with *Clostridium difficile* colitis. Eur J Clin Microbiol Infect Dis 21:736–738. 10.1007/s10096-002-0816-z 12479171 10.1007/s10096-002-0816-z

[15] McCroskey LM, Hatheway CL, Fenicia L, Pasolini B, Aureli P (1986) Characterization of an organism that produces type E botulinal toxin but which resembles *Clostridium butyricum* from the feces of an infant with type E botulism. J Clin Microbiol 23:201–202. 10.1128/jcm.23.1.201-202.1986 3517043 10.1128/jcm.23.1.201-202.1986 PMC268605

[16] Aureli P, Fenicia L, Pasolini B, Gianfranceschi M, McCroskey LM, Hatheway CL (1986) Two cases of type E infant botulism caused by neurotoxigenic *Clostridium butyricum* in Italy. J Infect Dis 154:207–211. 10.1093/infdis/154.2.207 3722863 10.1093/infdis/154.2.207

[17] Paisley JW, Lauer BA, Arnon SS (1995) A second case of infant botulism type F caused by *Clostridium baratii*. Pediatr Infect Dis J 14:912–914 8584326

[18] Halpin AL, Khouri JM, Payne JR, Nakao JH, Cronquist A, Kalas N, Mohr M, Osborne M, O’Dell S, Luquez C et al (2017) Type F infant botulism: investigation of recent clusters and overview of this exceedingly rare disease. Clin Infect Dis 66:S92–S94. 10.1093/cid/cix818 29293930 10.1093/cid/cix818

[19] Moodley A, Quinlisk P, Garvey A, Kalas N, Barash JR, Khouri JM (2015) Infant botulism caused by *Clostridium baratii* type F — Iowa, 2013. MMWR Morb Mortal Wkly Rep 64:400 25879901 PMC5779545

[20] Oguma K, Yokota K, Hayashi S, Takeshi K, Kumagai M, Itoh N, Tachi N, Chiba S (1990) Infant botulism due to *Clostridium botulinum* type C toxin. Lancet 336:1449–1450. 10.1016/0140-6736(90)93157-k 1978909 10.1016/0140-6736(90)93157-k

[21] Meyer KF, Dubovsky BJ (1922) The distribution of the spores of *B. botulinus* in the United States. IV J Infect Dis 31:559–594. 10.1093/infdis/31.6.559

[22] Meyer KF, Dubovsky BJ (1922) The distribution of the spores of *B. botulinus* in California. II J Infect Dis 31:541–555. 10.1093/infdis/31.6.541

[23] Meyer KF, Dubovsky BJ (1922) The occurrence of the spores of *B. botulinus* in Belgium, Denmark, England, The Netherlands and Switzerland. VI J Infect Dis 31:600–609. 10.1093/infdis/31.6.600

[24] Schoenholz P, Meyer KF (1922) The occurrence of the spores of *B. botulinus* in the Hawaiian Islands and China. VII J Infect Dis 31:610–613. 10.1093/infdis/31.6.610

[25] Dubovsky BJ, Meyer KF (1922) The distribution of the spores of *B. botulinus* in the territory of Alaska and the dominion of Canada. V J Infect Dis 31:595–599

[26] Brett MM, McLauchlin J, Harris A, O’Brien S, Black N, Forsyth RJ, Roberts D, Bolton FJ (2005) A case of infant botulism with a possible link to infant formula milk powder: evidence for the presence of more than one strain of *Clostridium botulinum* in clinical specimens and food. J Med Microbiol 54:769–776. 10.1099/jmm.0.46000-0 16014431 10.1099/jmm.0.46000-0

[27] Johnson EA, Tepp WH, Bradshaw M, Gilbert RJ, Cook PE, McIntosh EDG (2005) Characterization of *Clostridium botulinum* strains associated with an infant botulism case in the United Kingdom. J Clin Microbiol 43:2602–2607. 10.1128/JCM.43.6.2602-2607.2005 15956371 10.1128/JCM.43.6.2602-2607.2005 PMC1151885

[28] Spika JS, Shaffer N, Hargrett-Bean N, Collin S, MacDonald KL, Blake PA (1989) Risk factors for infant botulism in the United States. Am J Dis Child 143:828–832. 10.1001/archpedi.1989.02150190078026 2741856 10.1001/archpedi.1989.02150190078026

[29] Dabritz HA, Hill KK, Barash JR, Ticknor LO, Helma CH, Dover N, Payne JR, Arnon SS (2014) Molecular epidemiology of infant botulism in California and elsewhere, 1976–2010. J Infect Dis 210:1711–1722. 10.1093/infdis/jiu331 24924163 10.1093/infdis/jiu331

[30] Panditrao MV, Dabritz HA, Kazerouni NN, Damus KH, Meissinger JK, Arnon SS (2020) Seven-year case-control study in California of risk factors for infant botulism. J Pediatr 227:258-267.e8. 10.1016/j.jpeds.2020.07.014 32645406 10.1016/j.jpeds.2020.07.014

[31] Midura TF, Arnon SS (1976) Infant botulism. Identification of *Clostridium botulinum* and its toxins in faeces. Lancet 2:934–936. 10.1016/s0140-6736(76)90894-1 62164 10.1016/s0140-6736(76)90894-1

[32] Sugiyama H, Mills DC, Kuo L-C (1978) Number of *Clostridium botulinum* spores in honey. J Food Prot 41:848–850. 10.4315/0362-028X-41.11.848 30812109 10.4315/0362-028X-41.11.848

[33] Midura TF (1979) Laboratory aspects of infant botulism in California. Rev Infect Dis 1:652–655. 10.1093/clinids/1.4.652 399372 10.1093/clinids/1.4.652

[34] Chin J, Arnon SS, Midura TF (1979) Food and environmental aspects of infant botulism in California. Rev Infect Dis 1:693–697. 10.1093/clinids/1.4.693 399377 10.1093/clinids/1.4.693

[35] Arnon SS, Midura TF, Damus K, Thompson B, Wood RM, Chin J (1979) Honey and other environmental risk factors for infant botulism. J Pediatr 94:331–336. 10.1016/s0022-3476(79)80863-x 368301 10.1016/s0022-3476(79)80863-x

[36] Arnon SS, Damus K, Chin J (1981) Infant botulism: epidemiology and relation to sudden infant death syndrome. Epidemiol Rev 3:45–66. 10.1093/oxfordjournals.epirev.a036239 7030764 10.1093/oxfordjournals.epirev.a036239

[37] American Academy of Pediatrics Policy statement: honey and infant botulism. (1981) Pediatrics 32:5.

[38] Panditrao MV, Dabritz HA, Kazerouni NN, Damus KH, Meissinger JK, Arnon SS (2020) Descriptive epidemiology of infant botulism in California: The first 40 years. J Pediatr 227:247-257.e3. 10.1016/j.jpeds.2020.08.013 32800814 10.1016/j.jpeds.2020.08.013

[39] Hubtanen CN, Knox D, Sbimanuki H (1981) Incidence and origin of *Clostridium botulinum* spores in honey. J Food Prot 44:812–814. 10.4315/0362-028X-44.11.812 30856746 10.4315/0362-028X-44.11.812

[40] Hauschild A, Dodds K (1993) *Clostridium botulinum*: Ecology and control in foods. CRC Press, Boca Raton

[41] Nevas M, Lindström M, Hautamäki K, Puoskari S, Korkeala H (2005) Prevalence and diversity of *Clostridium botulinum* types A, B, E and F in honey produced in the Nordic countries. Int J Food Microbiol 105:145–151. 10.1016/j.ijfoodmicro.2005.04.007 16054259 10.1016/j.ijfoodmicro.2005.04.007

[42] Tølløfsrud PA, Kvittingen EA, Granum PE, Vøllo A (1998) Botulism in newborn infants. Tidsskr Nor Laegeforen 118:4355–4356 9889606

[43] Nevas M, Hielm S, Lindström M, Horn H, Koivulehto K, Korkeala H (2002) High prevalence of *Clostridium botulinum* types A and B in honey samples detected by polymerase chain reaction. Int J Food Microbiol 72:45–52. 10.1016/s0168-1605(01)00615-8 11843412 10.1016/s0168-1605(01)00615-8

[44] Kautter DA, Lilly T, Solomon HM, Lynt RK (1982) *Clostridium botulinum* spores in infant foods: A survey. J Food Prot 45:1028–1029. 10.4315/0362-028X-45.11.1028 30913614 10.4315/0362-028X-45.11.1028

[45] Lilly T, Rhodehamel EJ, Kautter DA, Solomon HM (1991) *Clostridium botulinum* spores in corn syrup and other syrups. J Food Prot 54:585–587. 10.4315/0362-028X-54.8.585 31051600 10.4315/0362-028X-54.8.585

[46] Waler JA (2000) Risk of infant botulism from corn syrup. Pediatr Infect Dis J 19:584 10877185 10.1097/00006454-200006000-00026

[47] Nakano H, Yoshikuni Y, Hashimoto H, Sakaguchi G (1992) Detection of *Clostridium botulinum* in natural sweetening. Int J Food Microbiol 16:117–121. 10.1016/0168-1605(92)90004-m 1445754 10.1016/0168-1605(92)90004-m

[48] Bianco MI, Lúquez C, de Jong LIT, Fernández RA (2008) Presence of *Clostridium botulinum* spores in Matricaria chamomilla (chamomile) and its relationship with infant botulism. Int J Food Microbiol 121:357–360. 10.1016/j.ijfoodmicro.2007.11.008 18068252 10.1016/j.ijfoodmicro.2007.11.008

[49] Bianco MI, Lúquez C, de Jong LIT, Fernández RA (2009) Linden flower (Tilia spp.) as potential vehicle of *Clostridium botulinum* spores in the transmission of infant botulism. Rev Argent Microbiol 41:232–236 20085187

[50] Hauschild AHW, Hilsheimer R, Weiss KF, Burke RB (1988) *Clostridium botulinum* in honey, syrups and dry infant cereals. J Food Prot 51:892–894. 10.4315/0362-028X-51.11.892 30991497 10.4315/0362-028X-51.11.892

[51] Report on minimally processed infant weaning foods and the risk of infant botulism. (2006) Food Standards Agency. https://acmsf.food.gov.uk/sites/default/files/mnt/drupal_data/sources/files/multimedia/pdfs/infantbotulismreport.pdf. Accessed September 25,2023.

[52] The Codex Alimentarius Commission (2008) RCP 66 - Code of Hygienic practice for powdered formulae for infants and young children. Food and Agriculture Organization of the United Nations. https://www.fao.org/input/download/standards/11026/CXP_066e.pdf. Accessed September 25, 2023.

[53] Usefulness of testing for *Clostridium botulinum* in powdered infant formula and dairy-based ingredients for infant formula. (2014) International Commission on Microbiological Specifications for Foods. International Union of Microbiological Societies. https://www.icmsf.org/wp-content/uploads/2018/02/ICMSF_Infant_Formula_Testing_Revision1-20140117.pdf. Accessed September 25, 2023.

[54] Barash JR, Hsia JK, Arnon SS (2010) Presence of soil-dwelling clostridia in commercial powdered infant formulas. J Pediatr 156:402–408. 10.1016/j.jpeds.2009.09.072 20004414 10.1016/j.jpeds.2009.09.072

[55] Dong YP, Wang W, Jiang T, Xu J, Han CH, Yan SF, Fanning S, Li Y, Ma XC, Zhang D et al (2017) Molecular and epidemiological characterization of infant botulism in Beijing, China. Biomed Environ Sci 30:460–464. 10.3967/bes2017.061 28705271 10.3967/bes2017.061

[56] Hauschild AHW, Aris BJ, Hilsheimer R (1975) *Clostridium botulinum* in marinated products. Can Inst Food Sci Technol J 8:84–87. 10.1016/S0315-5463(75)73727-6

[57] Lilly T, Solomon HM, Rhodehamel EJ (1996) Incidence of *Clostridium botulinum* in vegetables packaged under vacuum or modified atmosphere. J Food Prot 59:59–61. 10.4315/0362-028X-59.1.59 31158955 10.4315/0362-028X-59.1.59

[58] Braconnier A, Broussolle V, Perelle S, Fach P, Nguyen-The C, Carlin F (2001) Screening for *Clostridium botulinum* type A, B, and E in cooked chilled foods containing vegetables and raw material using polymerase chain reaction and molecular probes. J Food Prot 64:201–207. 10.4315/0362-028x-64.2.201 11271768 10.4315/0362-028x-64.2.201

[59] Sevenier V, Delannoy S, André S, Fach P, Remize F (2012) Prevalence of *Clostridium botulinum* and thermophilic heat-resistant spores in raw carrots and green beans used in French canning industry. Int J Food Microbiol 155:263–268. 10.1016/j.ijfoodmicro.2012.02.009 22405945 10.1016/j.ijfoodmicro.2012.02.009

[60] Pernu N, Keto-Timonen R, Lindström M, Korkeala H (2020) High prevalence of *Clostridium botulinum* in vegetarian sausages. Food Microbiol 91:103512. 10.1016/j.fm.2020.103512 32539985 10.1016/j.fm.2020.103512

[61] Burke GS (1919) The occurrence of *Bacillus botulinus* in nature. J Bacteriol 4:541–553 16558851 10.1128/jb.4.5.541-553.1919 PMC378819

[62] Hielm S, Hyytiä E, Andersin AB, Korkeala H (1998) A high prevalence of *Clostridium botulinum* type E in finnish freshwater and Baltic sea sediment samples. J Appl Microbiol 84:133–137. 10.1046/j.1365-2672.1997.00331.x 15244068 10.1046/j.1365-2672.1997.00331.x

[63] Harris RA, Blondin-Brosseau M, Levesque C, Rasmussen PE, Beauchemin S, Austin JW (2023) Viable *Clostridium botulinum* spores not detected in the household dust of major Canadian cities. Epidemiol Infect. 10.1017/S0950268823001474 37675600 10.1017/S0950268823001474 PMC10548537

[64] Long SS, Gajewski JL, Brown LW, Gilligan PH (1985) Clinical, laboratory, and environmental features of infant botulism in Southeastern Pennsylvania. Pediatrics 75:935–941 3887319

[65] Smith LD (1978) The occurrence of *Clostridium botulinum* and *Clostridium tetani* in the soil of the United States. Health Lab Sci 15:74–80 355208

[66] Leclair D, Farber JM, Doidge B, Blanchfield B, Suppa S, Pagotto F, Austin JW (2013) Distribution of *Clostridium botulinum* type E strains in nunavik, Northern Quebec, Canada. Appl Environ Microbiol 79:646–654. 10.1128/AEM.05999-11 23160120 10.1128/AEM.05999-11 PMC3553760

[67] Parry EW (1946) Prevalence of *Clostridium botulinum* in soils of central New York state. Food Res 11:203–209. 10.1111/j.1365-2621.1946.tb16345.x 20989616 10.1111/j.1365-2621.1946.tb16345.x

[68] Huss HH (1980) Distribution of *Clostridium botulinum*. Appl Environ Microbiol 39:764–769 6990867 10.1128/aem.39.4.764-769.1980 PMC291416

[69] Lúquez C, Bianco MI, de Jong LIT, Sagua MD, Arenas GN, Ciccarelli AS, Fernández RA (2005) Distribution of botulinum toxin-producing clostridia in soils of Argentina. Appl Environ Microbiol 71:4137–4139. 10.1128/AEM.71.7.4137-4139.2005 16000834 10.1128/AEM.71.7.4137-4139.2005 PMC1168975

[70] Xin W, Huang Y, Ji B, Li P, Wu Y, Liu J, Wang X, Yang H, Kang L, Gao S et al (2019) Identification and characterization of clostridium botulinum strains associated with an infant botulism case in China. Anaerobe 55:1–7. 10.1016/j.anaerobe.2018.06.015 30401636 10.1016/j.anaerobe.2018.06.015

[71] Saraiva M, Campos Cunha I, Costa Bonito C, Pena C, Toscano MM, Teixeira Lopes T, Sousa I, Calhau MA (2012) First case of infant botulism in Portugal. Food Control 26:79–80. 10.1016/j.foodcont.2012.01.010

[72] Kobayashi T, Haginoya K, Morimoto T, Hatakeyama T, Tsuchiya S (2014) A case of infant botulism infection due to consumption of untreated well-water. J Pediatr 164:931–933. 10.1016/j.jpeds.2013.11.044 24461790 10.1016/j.jpeds.2013.11.044

[73] Shelley EB, O’Rourke D, Grant K, McArdle E, Capra L, Clarke A, McNamara E, Cunney R, McKeown P, Amar CF et al (2015) Infant botulism due to *C. butyricum* type E toxin: a novel environmental association with pet terrapins. Epidemiol Infect 143:461–469. 10.1017/S0950268814002672 25306863 10.1017/S0950268814002672 PMC9507071

[74] Goldberg B, Danino D, Levinsky Y, Levy I, Straussberg R, Dabaja-Younis H, Guri A, Almagor Y, Tasher D, Elad D et al (2023) Infant botulism, Israel, 2007–2021. Emerg Infect Dis 29:235–241. 10.3201/eid2902.220991 36692296 10.3201/eid2902.220991 PMC9881770

[75] Stark PL, Lee A (1982) The microbial ecology of the large bowel of breastfed and formula-fed infants during the first year of life. J Med Microbiol 15:189–203. 10.1099/00222615-15-2-189 7143428 10.1099/00222615-15-2-189

[76] Palmer C, Bik EM, DiGiulio DB, Relman DA, Brown PO (2007) Development of the human infant intestinal microbiota. PLoS Biol 5:e177. 10.1371/journal.pbio.0050177 17594176 10.1371/journal.pbio.0050177 PMC1896187

[77] Rodríguez M, Stanton R, Kober J, Avershina R, Narbad J et al (2015) The composition of the gut microbiota throughout life, with an emphasis on early life. Microb Ecol Health Dis. 10.3402/mehd.v26.26050 25651996 10.3402/mehd.v26.26050 PMC4315782

[78] Derrien M, Alvarez A, de Vos WM (2019) The gut microbiota in the first decade of life. Trends Microbiol 27:997–1010. 10.1016/j.tim.2019.08.001 31474424 10.1016/j.tim.2019.08.001

[79] Moberg LJ, Sugiyama H (1979) Microbial ecological basis of infant botulism as studied with germfree mice. Infect Immun 25:653–657 385503 10.1128/iai.25.2.653-657.1979 PMC414494

[80] Wells CL, Sugiyama H, Bland SE (1982) Resistance of mice with limited intestinal flora to enteric colonization by *Clostridium botulinum*. J Infect Dis 146:791–796. 10.1093/infdis/146.6.791 6754827 10.1093/infdis/146.6.791

[81] Burr DH, Sugiyama H (1982) Susceptibility to enteric botulinum colonization of antibiotic-treated adult mice. Infect Immun 36:103–106 7042567 10.1128/iai.36.1.103-106.1982 PMC351190

[82] Thompson JA, Glasgow LA, Warpinski JR, Olson C (1980) Infant botulism: clinical spectrum and epidemiology. Pediatrics 66:936–942 7005856

[83] Shirey TB, Dykes JK, Lúquez C, Maslanka SE, Raphael BH (2015) Characterizing the fecal microbiota of infants with botulism. Microbiome 3:54. 10.1186/s40168-015-0119-0 26593441 10.1186/s40168-015-0119-0 PMC4655454

[84] Lupp C, Robertson ML, Wickham ME, Sekirov I, Champion OL, Gaynor EC, Finlay BB (2007) Host-mediated inflammation disrupts the intestinal microbiota and promotes the overgrowth of Enterobacteriaceae. Cell Host Microbe 2:119–129. 10.1016/j.chom.2007.06.010 18005726 10.1016/j.chom.2007.06.010

[85] Douillard FP, Derman Y, Jian C, Korpela K, Saxén H, Salonen A, de Vos WM, Korkeala H, Lindström M (2024) Case report: aberrant fecal microbiota composition of an infant diagnosed with prolonged intestinal botulism. Gut Pathog 16:20. 10.1186/s13099-024-00614-y 38581020 10.1186/s13099-024-00614-y PMC10996148

[86] Picard C, Fioramonti J, Francois A, Robinson T, Neant F, Matuchansky C (2005) Review article: bifidobacteria as probiotic agents—physiological effects and clinical benefits. Aliment Pharmacol Ther 22:495–512. 10.1111/j.1365-2036.2005.02615.x 16167966 10.1111/j.1365-2036.2005.02615.x

[87] Sullivan NM, Mills DC, Riemann HP, Arnon SS (2011) Inhibition of growth of *Clostridium botulinum* by intestinal microflora isolated from healthy infants. Microb Ecol Heal Dis. 10.3402/mehd.v1i3.7413

[88] Lam TI, Tam CC, Stanker LH, Cheng LW (2016) Probiotic microorganisms inhibit epithelial cell internalization of botulinum neurotoxin serotype A. Toxins (Basel) 8:377. 10.3390/toxins8120377 27999281 10.3390/toxins8120377 PMC5198571

[89] Fernandez RA, Carbone ML, Sanchez ML, Pareja Virtudes, de Jong LIT, Bianco MI (2013) Interference of the developing and toxin production of *Clostridium botulinum* by *Lactobacillus paracasei* subspecies *paracasei*. International Journal of Sciences 2.

[90] Iqbal Z, Ahmed S, Tabassum N, Bhattacharya R, Bose D (2021) Role of probiotics in prevention and treatment of enteric infections: a comprehensive review. 3 Biotech. 10.1007/s13205-021-02796-7 33968585 10.1007/s13205-021-02796-7 PMC8079594

[91] Barash JR, Castles JB, Arnon SS (2018) Antimicrobial susceptibility of 260 *Clostridium botulinum* type A, B, Ba, and Bf strains and a neurotoxigenic *Clostridium baratii* type F strain isolated from California infant botulism patients. Antimicrob Agents Chemother 62:1594. 10.1128/AAC.01594-18 10.1128/AAC.01594-18 PMC6256804 30275093

[92] Arnon SS (1980) Infant botulism. Annu Rev Med 31:541–560. 10.1146/annurev.me.31.020180.002545 6772092 10.1146/annurev.me.31.020180.002545

[93] Nevas M, Lindström M, Virtanen A, Hielm S, Kuusi M, Arnon SS, Vuori E, Korkeala H (2005) Infant botulism acquired from household dust presenting as sudden infant death syndrome. J Clin Microbiol 43:511–513. 10.1128/JCM.43.1.511-513.2005 15635031 10.1128/JCM.43.1.511-513.2005 PMC540168

[94] Midura TF (1996) Update: infant botulism. Clin Microbiol Rev 9:119–125. 10.1128/CMR.9.2.119 8964030 10.1128/cmr.9.2.119 PMC172885

[95] Hatheway CL, McCroskey LM (1987) Examination of feces and serum for diagnosis of infant botulism in 336 patients. J Clin Microbiol 25:2334–2338. 10.1128/jcm.25.12.2334-2338.1987 3323228 10.1128/jcm.25.12.2334-2338.1987 PMC269483

[96] Dubovsky BJ, Meyer KF (1922) An experimental study of the methods available for the enrichment, demonstration and isolation of *B. botulinus* in specimens of soil and its products, in suspected food, in clinical and in necropsy material. I J Infect dis 31:501–540. 10.1093/infdis/31.6.501

[97] Dabritz HA, Payne JR, Khouri JM (2023) Duration of fecal excretion of *Clostridium botulinum* and botulinum neurotoxin in children recovering from infant botulism. J Pediatr. 10.1016/j.jpeds.2023.113396 37004956 10.1016/j.jpeds.2023.113396

